# Hydrogen generation by reaction of Si nanopowder with neutral water

**DOI:** 10.1007/s11051-017-3873-z

**Published:** 2017-05-16

**Authors:** Yuki Kobayashi, Shinsuke Matsuda, Kentaro Imamura, Hikaru Kobayashi

**Affiliations:** The Institute of Scientific and Industrial Research, Osaka University, 8-1 Mihogaoka, Osaka, Ibaraki 567-0047 Japan

**Keywords:** Hydrogen-rich water, SiO_2_, Hydroxide ion, Hydroxyl radical, Medical applications

## Abstract

Si and its oxide are nonpoisonous materials, and thus, it can be taken for medical effects. We have developed a method of generation of hydrogen by use of reactions of Si nanopowder with water in the neutral pH region. Si nanopowder is fabricated by the simple bead milling method. Si nanopowder reacts with water to generate hydrogen even in cases where pH is set at the neutral region between 7.0 and 8.6. The hydrogen generation rate strongly depends on pH and in the case of pH 8.0, ∼55 ml/g hydrogen which corresponds to that contained in approximately 3 L saturated hydrogen-rich water is generated in 1 h. The reaction rate for hydrogen generation greatly increases with pH, indicating that the reacting species is hydroxide ions. The change of pH after the hydrogen generation reaction is negligibly low compared with that estimated assuming that hydroxide ions are consumed by the reaction. From these results, we conclude the following reaction mechanism: Si nanopowder reacts with hydroxide ions in the rate-determining reaction to form hydrogen molecules, SiO_2_, and electrons in the conduction band. Then, generated electrons are accepted by water molecules, resulting in production of hydrogen molecules and hydroxide ions. The hydrogen generation rate strongly depends on the crystallite size of Si nanopowder, but not on the size of aggregates of Si nanopowder. The present study shows a possibility to use Si nanopowder for hydrogen generation in the body in order to eliminate hydroxyl radicals which cause various diseases.

**Electronic supplementary material:**

The online version of this article (doi:10.1007/s11051-017-3873-z) contains supplementary material, which is available to authorized users.

## Introduction

Hydrogen and hydrogen-rich water attract much interest because of its excellent property to eliminate hydroxyl radicals in our bodies (Ohsawa et al. 2007; Iuchi et al. 2016). It is reported that hydrogen-rich water (or hydrogen) possesses an effect to prevent various diseases such as cancer cell proliferation (Kagawa et al. 2012), memory deterioration (Nagata et al. 2009), Parkinson’s disease (Yoritaka et al. 2013; Fu et al. 2009), Alzheimer’s disease (Li et al. 2010), diabetes (Kamimura et al. 2011), obesity (Kamimura et al. 2011), atopic dermatitis (Yoon et al. 2014), cutaneous senility (Kato et al. 2012), etc.

Si and SiO_2_ (i.e., product formed by the reaction of Si with water to generate hydrogen) are both nonpoisonous, and thus, Si nanopowder can be taken to generate hydrogen in the body. Generated hydrogen is absorbed in the digestive organ and circulates in the vascular system of whole body.

The conventional method to produce hydrogen-rich water utilizes water electrolysis. Disadvantage of this method is needs of electrolysis apparatuses and electricity. Another disadvantage is that the hydrogen concentration easily decreases by diffusion to air. Moreover, the saturated hydrogen concentration of hydrogen-rich water is only 1.6 ppm, and 1 L hydrogen-rich water includes only 19 mL hydrogen gas at maximum.

Litvinenko et al. (2010) have reported that porous Si produced by stain etching reacts with water in the presence of NH_3_, leading to generation of hydrogen molecules. Bahruji et al. (2009) have shown that irradiation of UV light on Si nanoparticles in deionized water generates hydrogen, resulting from oxidation of Si nanoparticles. Erogbogbo et al. (2013) have achieved a high hydrogen evolution rate by the reaction of nano-sized Si with water having high pH. These studies aimed at hydrogen generation with high rates for energy application, e.g., fuel cells, and these methods using strong alkaline solutions or UV irradiation are not applicable to internal hydrogen generation for medical use.

We have recently developed a simple fabrication method of Si nanopowder using the bead milling method (Maeda et al. 2014; Matsumoto et al. 2014; Matsumoto et al. 2016; Imamura et al. 2016) and shown that fabricated Si nanopowder exhibits visible light photoluminescence due to band-gap widening resulting from the quantum confinement effect (Matsumoto et al. 2016). Although Si bulk does not strongly react with water to generate hydrogen, Si nanopowder easily reacts with it especially in the case of high pH (Erogbogbo et al. 2013; Imamura et al. 2016). At pH of 13, for example, the hydrogen evolution rate reaches 580 mL/min·g, and the total hydrogen evolution volume is 1.44 L/g (Imamura et al. 2016).

In the present study, it is shown that Si nanopowder reacts with water having neutral pH range between 7.0 and 8.6 to generate hydrogen (or hydrogen-rich water). The present study shows a possibility to generate hydrogen in the human body to eliminate hydroxyl radicals (·OH) which are the possible cause for various diseases such as diabetic vascular disease (Kajiyama et al. 2008), Alzheimer’s disease (Maurizi 2001), Parkinson’s disease (Ihara et al. 1999), Huntington’s disease (Charvin et al. 2005), cataract (Garner et al. 2000), cutaneous aging (Cadenas and Davies 2000; Pillai et al. 2005), etc.

## Experimental

Si nanopowder was produced from Si powder (Koujundo Chemical Laboratory Si 3 N Powder ca. 5 μm) by use of the bead milling method. Si nanopowder was fabricated using the one-step milling method with 0.5-mm diameter zirconia beads for 4 h or using the two-step milling method with 0.5-mm diameter zirconia beads for 4 h and then with 0.3-mm diameter zirconia beads for 4 h. For some specimens, etching with 5 wt% hydrofluoric acid (HF) solutions was carried out to remove a silicon oxide layer on Si nanopowder. Si nanopowder after the HF treatment was hydrophobic due to surface Si–H bonds (Jakob et al. 1992; Higashi et al. 1991). Si nanopowder was immersed in ethanol to make the surface hydrophilic and thus to promote surface reaction with water. Si nanopowder of 10 mg with a small amount of ethanol was immersed in 30 mL water with pH adjusted between 7.0 and 8.6 by addition of borate buffer. In some cases, water with pH 9.0 was used for hydrogen generation.

The hydrogen concentration in water was detected by use of a TOA DKK-TOA DH-35A potable dissolved hydrogen-meter, and the generated hydrogen volume at 20 °C was estimated from the hydrogen concentration. X-ray photoelectron spectroscopy (XPS) spectra were recorded using a KRATOS AXIS-165x spectrometer with an Mg-Kα radiation source.

## Results and discussion

**Fig. 1:**
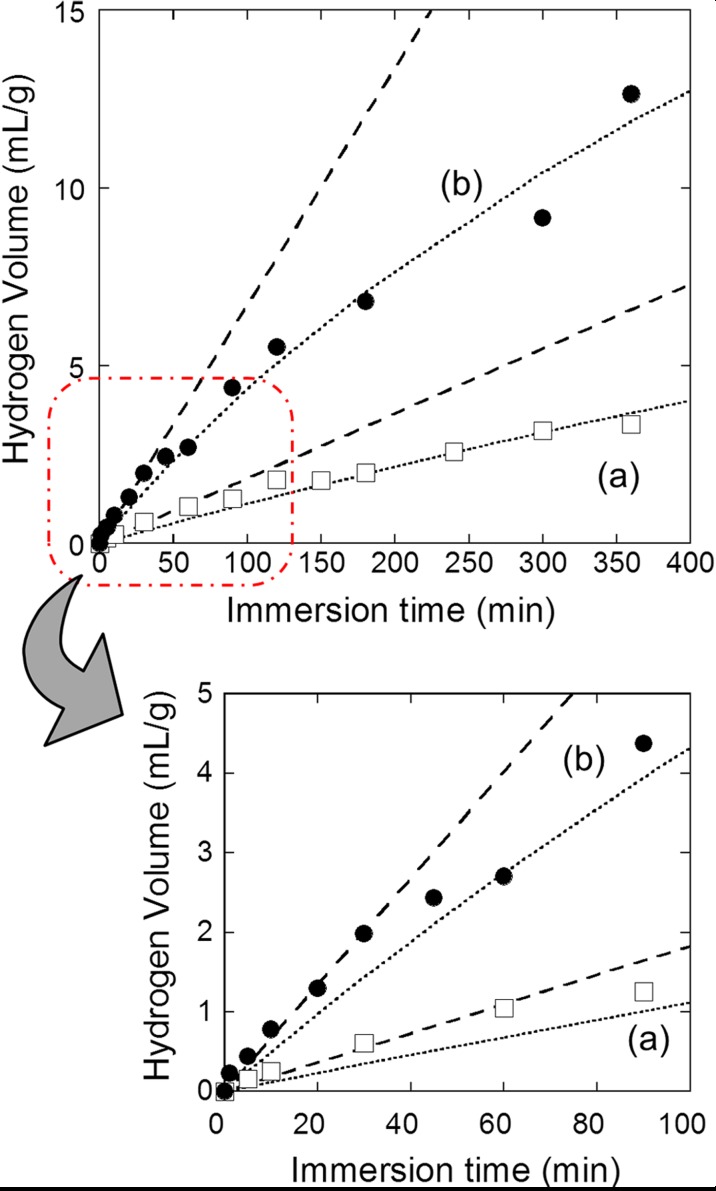
Generated hydrogen volume vs the immersion time of Si nanopowder under the following conditions in ultrapure water: **a** as-prepared; **b** after etching with an HF solution. Si nanopowder was produced by use of the one-step milling method with 0.5-mm diameter zirconia beads. Generated hydrogen volume was estimated from the hydrogen concentration in water. The *dashed and dotted lines* show the calculated relationship for the reaction-limited and migration-limited mechanisms, respectively. The lower portion shows the enlarged figure of the early stage of hydrogen generation from Si nanopowder

Figure 1 shows the volume of hydrogen generated by the reaction with ultrapure water vs the reaction time for Si nanopowder produced by the one-step milling method with (plot b) and without (plot a) the HF treatment. With HF etching of Si nanopowder, the generated hydrogen volume increased much rapidly with time, and that measured after 6 h reaction became ∼4 times higher than that without HF etching. Both the plots were almost linear with the reaction time.

**Fig. 2:**
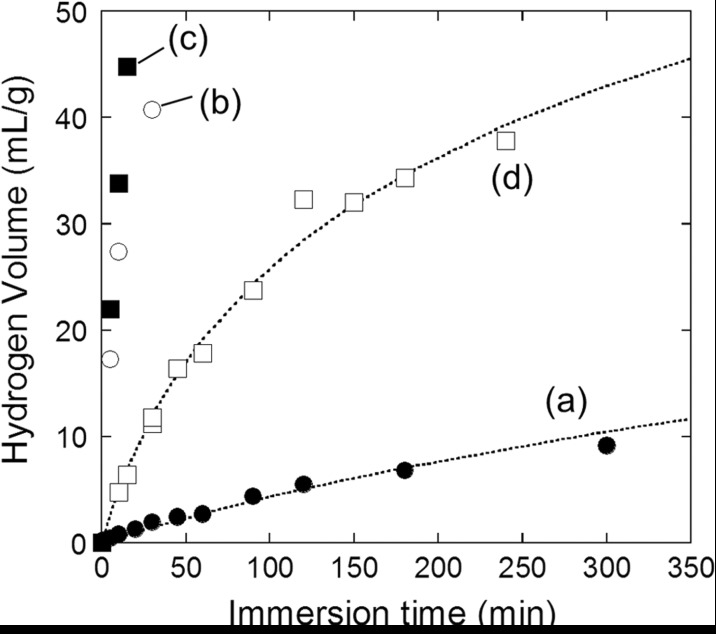
Generated hydrogen volume vs the immersion time of Si nanopowder in the following solutions: **a** ultrapure water; **b** water with pH 8.0; **c** water with pH 8.6; **d** tap water with pH 7.1∼7.4. Si nanopowder was produced by use of the one-step milling method with 0.5-mm diameter zirconia beads. Si nanopowder was etched with an HF solution to remove silicon oxide before immersion

Figure 2 shows the hydrogen volume produced by the reactions of Si nanopowder with water having various pH values. The generated hydrogen volume for pH 8.0 (plot b) solutions increased much faster than that for ultrapure water (plot a). The hydrogen generation rate was further increased by an increase of pH to 8.6 (plot c). Hydrogen generation of ∼55 mL/g was achieved in 60 min for water with pH 8.0 and 25 min for that with pH 8.6. Hydrogen generation also proceeded by the reaction with tap water (pH = 7.1∼7.4) (plot d). It is noted that 55 mL hydrogen corresponded to that included in approximately 3 L saturated hydrogen-rich water although only 3.2% Si reacted for hydrogen generation. It should also be noted that pH of pancreatic juice is in the range between 7.6 and 8.9, and therefore, the present result shows a possibility that Si nanopowder can generate hydrogen in bowels where the absorption efficiency is high.

The above result clearly shows that Si nanopowder reacts with water having neutral pH, resulting in hydrogen evolution. The hydrogen generation ratio for the solution with pH of 8.0 (plot b) in the initial reaction stage between 0 and 5 min is approximately 40 times higher than that for ultrapure water (plot a). The ratio for shorter initial period is likely to be higher because the reaction at 5 min for the pH 8.0 solution may already be retarded by the formation of an oxide layer. This result indicates that the reacting species is hydroxide ions (OH^−^), but not water molecules. Therefore, the reaction formulae are written as

1 $$ Si+2{OH}^{-}\to {SiO}_2+{H}_2+2 e $$

2 $$ {2 H}_2 O+2 e\to {H}_2+2{OH}^{-} $$

and the total reaction formula is expressed as

3 $$ Si+2{H}_2 O\to {SiO}_2+2{H}_2 $$

In cases where reaction (1) is the rate-determining step, the reaction rate is proportional to the square of the concentration of OH^−^ ions because of the second-order reaction. Reaction (1) generates electrons in the oxide conduction band, and they are accepted by water molecules, resulting in water decomposition, and generation of hydrogen molecules and OH^−^ ions (reaction (2)). Therefore, reaction (1) followed by reaction (2) does not change the concentration of OH^−^ ions. To confirm this conclusion, we have performed the following experiment: Using a pH 9.0 solution, pH of the solution was found to change to 8.6 after hydrogen evolution. Assuming that hydrogen evolution proceeded by consuming OH^−^ ions, the equivalent HCl solution was added to the pH 9.0 solution, and in this case, pH was observed to change to 6.6. This result demonstrates that the hydrogen generation reaction does not consume OH^−^ ions. The slight decrease in pH from 9.0 to 8.6 was most likely to be caused by dissolution of CO_2_ in the air.

**Fig. 3:**
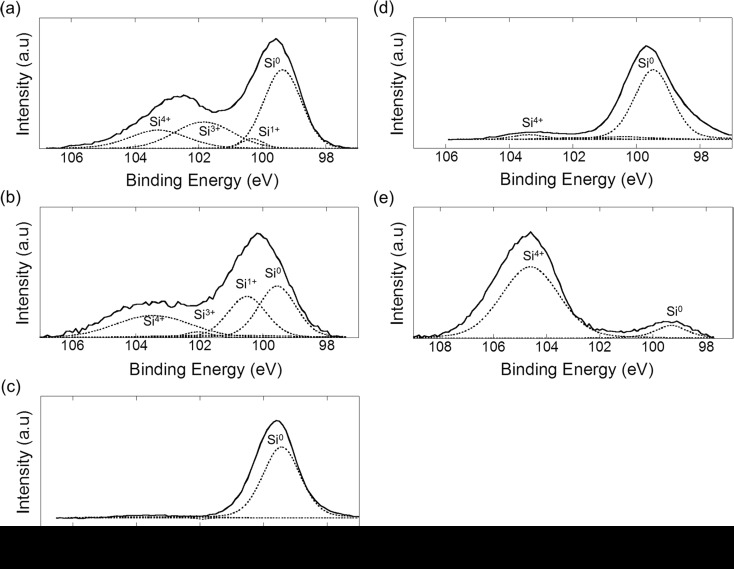
XPS spectra in the Si 2p region for Si nanopowder with the following conditions: **a** as-prepared Si nanopowder; **b** after reaction of as-prepared Si nanopowder with ultrapure water for 360 min; **c** after etching with an HF solution; **d** after reaction of HF-etched Si nanopowder with ultrapure water for 24 h; **e** after reaction of HF-etched Si nanopowder with water having pH 8.0 for 24 h. The *dotted lines* indicate components of unreacted Si (Si^0^), and Si atoms to which one (Si^1+^), three (Si^3+^), and four oxygen (Si^4+^) atoms are bound each. For the deconvoluted peaks, only the Si 2p_3/2_ components are depicted. Photoelectrons were collected in the surface-normal direction

Figure 3 shows the XPS spectra in the Si 2p region of Si nanopowder. For as-prepared Si nanopowder (spectrum a), two peaks were observed at 99.6 and 102.6 eV, attributable to Si and silicon oxide, respectively (Himpsel et al. 1988; Kobayashi et al. 1991). Taking into account that each Si 2p peak contained two components due to Si 2p_3/2_ and 2p_1/2_ having the same widths and the intensity ratio of 2:1 separated by 0.612 eV (Bozek et al. 1990), the observed XPS spectra were deconvoluted, and only the Si 2p_3/2_ components were shown by the dotted line in the spectra. The Si 2p_3/2_ components at 100.3, 101.9, and 103.3 eV are most probably attributable to Si atoms to which one, three, and four oxygen atoms (hereafter, denoted as Si^+^, Si^3+^, and Si^4+^, respectively) are bound each (Himpsel et al. 1988; Kobayashi et al. 1991). The intensity of the Si^2+^ species was negligibly low. After reaction of as-prepared Si nanopowder with ultrapure water for 360 min (spectrum b), the intensities of the peaks due to Si^4+^ and Si^+^ increased while those due to Si^3+^ and Si^0^ decreased.

After HF etching of as-prepared Si nanopowder (spectrum c), the peak due to silicon oxide disappeared almost completely, indicating removal of oxide. After reaction of HF-etched Si nanopowder with ultrapure water (spectrum d) and water having pH 8.0 for 24 h (spectrum e), the oxide peak was observed. In the latter case, the peak due to oxide was much stronger than the former case, and deconvolution showed that oxide consisted of Si^4+^ species (i.e., SiO_2_) without suboxide species.

For elucidation of the rate-determining step (i.e., surface reaction or migration of OH^−^ ions through silicon oxide), the thickness of a silicon oxide layer on Si nanopowder should be determined (Deal and Grove 1965), which can be done from analysis of the observed XPS spectra, as explained below. The size of Si nanopowder was estimated from the analysis of the XRD data assuming spherical shape. However, the XPS spectra cannot be analyzed assuming the spherical shape, and instead, a cylindrical shape which is reported to express the spherical shape reasonably well for estimation of the overlayer thickness (Renault et al. 2003) is adopted. Using the area intensity ratio of the oxide peak, *I*_*oxide*_, to that of the unreacted Si peak, *I*_*Si*_, and assuming the cylindrical structure with the radius, *R*, and the height, *H*, the oxide thickness, *l*_*oxide*_, can be estimated using the following equation (Renault et al. 2003):

4 $$ \frac{I_{oxide}}{I_{Si}}=\frac{n_{oxide}{\sigma}_{oxide}{\lambda}_{oxide}}{n_{Si}{\sigma}_{Si}{\lambda}_{Si}}\times \frac{{\left( R-{l}_{oxide}\right)}^2\left[1-\mathit{\exp}\left(\frac{l_{oxide}}{\lambda_{oxide}}\right)\right]+{l}_{oxide}\left(2 R-{l}_{oxide}\right)\left[1-\mathit{\exp}\left(-\frac{H}{\lambda_{oxide}}\right)\right]}{{\left( R-{l}_{oxide}\right)}^2\mathit{\exp}\left(-\frac{l_{oxide}}{\lambda_{oxide}}\right)\left[1-\mathit{\exp}\left(-\frac{H-{l}_{oxide}}{\lambda_{Si}}\right)\right]} $$

where *n*, σ, and λ are the number density of Si atoms, the photoemission cross-section, and the photoelectron mean free path, respectively, the subscripts, *oxide* and *Si*, denote values for silicon oxide and Si, respectively. Hereafter, the oxide thickness is estimated assuming *H* = *R* and *R* = 11.7 nm (i.e., the average size of Si nanopowder determined from the width of the XRD peaks). The detailed information concerning the size of Si nanopowder is given in the supplementary material. Using Eq. (4), and adopting 2.5 nm for *λ*_*Si*_, 2.9 nm for *λ*_*oxide*_ (Kobayashi et al. 2003) and 1.1 for *σ*_*oxide*_/*σ*_*Si*_ (Hochella and Carim 1988), the oxide thickness on as-prepared Si nanopowder (cf. Fig. 3a) is estimated to be 1.6 nm. In this estimation, the peaks due to Si^4+^ and Si^3+^ are regarded as oxide, but not Si^+^. The intensity of the Si^2+^ peak was negligibly low.

It is confirmed from the XPS measurements that after the hydrogen evolution reaction stopped, a 4.8 nm oxide layer was formed on Si nanopowder (cf. Fig. 3e). This result indicates that the 4.8 nm oxide layer acts as complete migration barrier for OH^−^ ions.

It should be noted that until the formation of the 4.8 nm SiO_2_ layer, ~300 mL/g hydrogen was observed to be generated, which corresponded to reaction of ~18% Si for hydrogen generation. This amount of hydrogen corresponds to that contained in ∼16 L saturated hydrogen-rich water. Using Si nanopowder, such a large amount of hydrogen can be generated in our body.

Assuming spherical shape for Si nanopowder, the volume of generated hydrogen, *V*_*H*_, is given by

5 $$ {V}_H={a}_1\left[{r}_0^3-{\left({r}_0-\varDelta r(t)\right)}^3\right] $$

where *r*_0_ is the initial radius of Si nanopowder, ∆*r* is the decrease of the radius of Si nanopowder consumed for oxide formation, *t* is the reaction time, and *a*_1_ is the constant. Considering that the volume of consumed Si for oxide formation is 0.46 times that of formed oxide (Sze and Ng 2006), we have

6 $$ {r}_0^3-{\left[{r}_0-\varDelta r(t)\right]}^3=0.46\left[{\left\{{r}_0-\varDelta r(t)+{l}_{oxide}(t)\right\}}^3-{\left\{{r}_0-\varDelta r(t)\right\}}^3\right] $$

In cases where the reaction at the Si surface is the rate-determining step (Deal and Grove 1965), the silicon oxide thickness, *l*_*oxide*_, is given by

7 $$ {l}_{oxide}={a}_2 t $$

where *a*_2_ is the constant. Using Eqs. (5), (6), and (7), the relationship between the generated hydrogen volume and the reaction time can be calculated, and the calculated result is given by the dashed lines in Fig. 1.

When the oxide thickness exceeds a certain value, movement of OH^−^ ions (i.e., migration) becomes rate-determining. In cases where inward migration of negative ions is the rate-determining-step (Eley and Wilkinson 1960), the oxide thickness, *l*_*oxide*_, is given by

8 $$ {l}_{oxide}=\frac{kT}{a_3}\mathit{\ln}\frac{a_3{a}_4\left( t+{t}_0\right)}{kT}-\frac{W}{a_3} $$

where *a*_3_ and *a*_4_ are the constants, *W* is the activation energy for migration of OH^−^ ions in the absence of silicon oxide, and *t*_0_ is written as

9 $$ {t}_0=\frac{kT}{a_3{a}_4}\mathit{\exp}\left[\frac{a_4}{kT}\left\{{l}_{oxide}(0)+\frac{W}{a_4}\right\}\right] $$

Inserting *l*_*oxide*_ obtained from Eqs. (8) and (9) into Eq. (6), the relationship between the generated hydrogen volume, *V*_*H*_, and the reaction time, *t*, can be calculated, and they are shown by the dotted lines in Figs. 1 and 2.

The calculated curves for the migration-limited case shown by the dotted lines in Figs. 1 and 2 well express the experimental results. For the migration-limited process, constants *a*_3_ and *a*_4_ in Eqs. (8) and (9) determine the generated hydrogen volume. Using the *a*_3_ and *a*_4_ values obtained from the curve fitting, the generated hydrogen volume after the reaction for 24 h is estimated to be 26.5 mL/g. The generated hydrogen volume can also be estimated from the thickness of the silicon oxide layer determined from the XPS spectrum (cf. Fig. 3d). Thus estimated hydrogen volume of 28.3 mL/g is in good agreement with that estimated from the curve fitting, which also verifies the migration-limited mechanism.

Plot b for Si nanopowder after HF-etching in the reaction time region up to 30 min deviates from the calculated curve for the migration-limited case while it is well expressed by the dashed line for the reaction-limited case (cf. enlarged figure of Fig. 1). HF etching removes silicon oxide almost completely. Therefore, migration of OH^−^ ions through silicon oxide proceeds smoothly, leading to the reaction-limited mechanism. The thickness of the SiO_2_ layer on HF-etched Si nanopowder after the reaction for 30 min is estimated to be 0.13 nm from the XPS spectrum for as-etched Si nanopowder and the generated hydrogen volume. This SiO_2_ thickness is in reasonable agreement with the Si–O bond length of 0.15∼0.16 nm, indicating the formation of approximately monolayer Si–O structure. Therefore, it can be concluded that the reaction at the Si/SiO_2_ interface is the rate-determining step until monolayer Si–O bonds are formed, and after the formation of monolayer Si–O bonds, migration of OH^−^ ions through SiO_2_ becomes rate-limiting. The small SiO_2_ thickness at the mechanism-changing point is due to the reaction at room temperature at which movement of oxidizing species is very slow in contrast to thermal oxidation of Si at temperatures higher than 800 °C.

In the case of Si nanopowder without HF etching, a 1.6 nm silicon oxide layer is present on the nanocrystalline Si surfaces (cf. Fig. 3a). Removal of the oxide layer by HF etching greatly increased the reaction rate (cf. Fig. 1a, b), indicating that the oxide layer retarded the reaction. In fact, the calculated curve for the reaction-limited process (the dashed line in Fig. 1), which curve is determined from the silicon oxide thickness, largely deviates from the experimental result shown in Fig. 1a. On the other hand, the calculated curve for the migration-limited mechanism shown by the dotted line can well express the experimental result. The XPS measurements show that for Si nanopowder without HF etching, the oxide thickness increased only slightly from 1.56 to 1.60 nm after reaction for 360 min (cf. Fig. 3b). It is noted that the logarithmic curve for the migration-limited mechanism in this narrow thickness region is almost linear.

In the case of the reaction with tap water (cf. Fig. 2d), the experimental result is well fitted by the calculated curve for the migration-limited mechanism. For the very initial reaction stage with the oxide thickness less than 0.13 nm (i.e., generated hydrogen volume less than 2.0 mL/g), the reaction is likely to be the rate-determining step, but due to the short period for the reaction-limited process, it was not clearly observed in plot d in Fig. 2. Therefore, it can be concluded that in the case of pH higher than 7.4, the hydrogen generation reaction follows the migration-limited kinetics in almost the whole reaction period.

**Fig. 4:**
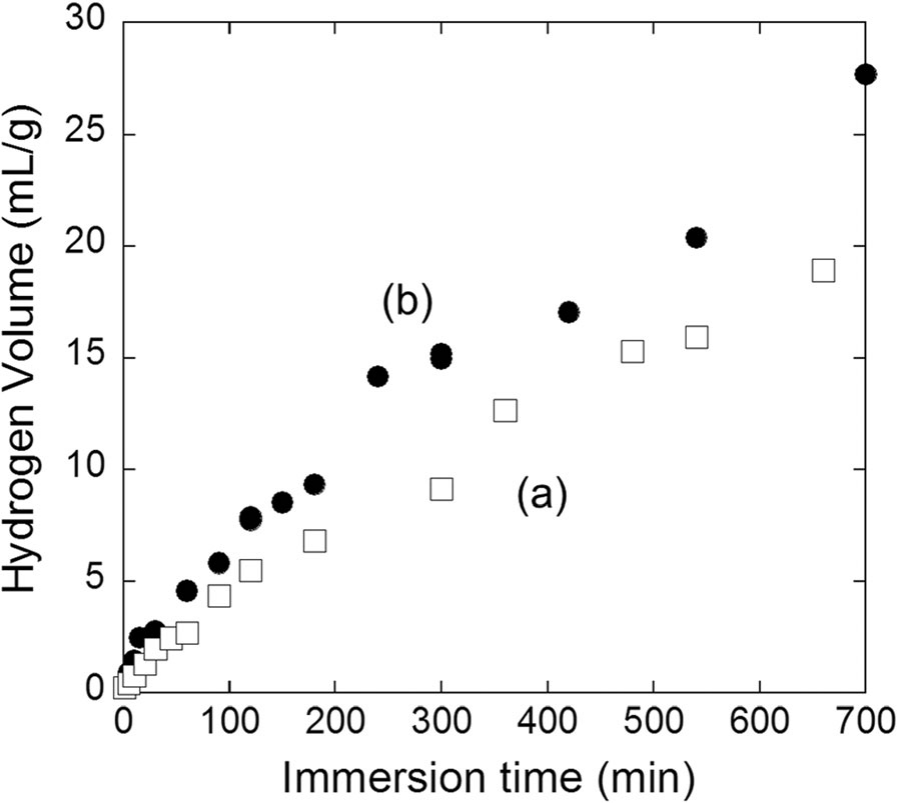
Generated hydrogen volume vs the time of immersion in ultrapure water for Si nanopowder produced by the following method: **a** one-step milling; **b** two-step milling. For one-step and two-step milling, 0.5-mm diameter zirconia beads and those plus 0.3-mm diameter zirconia beads were employed. Si nanopowders were treated with an HF solution before immersion

Figure 4 shows the volume of hydrogen generated by the reaction of Si nanopowder produced by the one-step (plot a) and two-step (plot b) milling methods with ultra-pure water. Both the plots were nearly linear, and the rate of an increase in the hydrogen volume for plot b in the initial hydrogen generation stage where the reaction was the rate-determining step was approximately 1.5 times of that for plot a.

The average diameters of Si crystallites for one-step and two-step milling determined from the XRD measurements (cf. Fig. S1 in supplementary material) are 23.4 and 13.8 nm, respectively, and the surface areas of the former and latter Si nanopowders are roughly estimated to be 110 and 190 m^2^/g, respectively. The ratio of the surface areas of 1.7 shows a reasonable agreement with the ratio of the hydrogen generation rates of 1.5. This result indicates that the reactivity of Si nanopowder is determined by the Si crystallite size. It should be noted that the surface areas estimated from the size of aggregated Si nanopowders are nearly the same for one-step and two-step milling, i.e., 12.8 and 13.4 m^2^/g, respectively (cf. Fig. S4 in supplementary material). The above result shows that the hydrogen generation rate strongly depends on the crystallite size of Si nanopowder, but it is nearly independent of the size of agglomerates.

The specific surface areas are determined to be 74 and 143 m^2^/g, respectively, using the nitrogen physisorption method. The estimated specific surface areas are ~40 m^2^/g smaller than the surface areas determined from the average crystallite sizes. This difference may be due to the presence of micropores (i.e., <2 nm) to which nitrogen molecules cannot enter to form a second nitrogen layer (physisorbed layer). Because of the smaller size of OH^−^ ions than a part of micropores, the ions may be able to enter them and the ratio of the effective surface areas between two-step and one-step milled Si nanopowders is likely to be smaller than the ratio of the specific surface areas determined by the BET method.

As explained above, it is clearly shown that Si nanopowder reacts with water in the neutral pH region to generate hydrogen. Since Si nanopowder and its oxide are nonpoisonous, Si nanopowder can be taken to generate hydrogen in the human body. No reaction proceeds in a stomach where pH is low (pH of gastric juices: 1.5∼2.0), while it reacts with OH^−^ ions in small intestine where pancreatic juice with pH in the range between 7.5 and 8.8 is injected and also absorption efficiency is high.

## Conclusion

We have shown that Si nanopowder reacts with water even with the neutral pH range between 7.0 and 8.6, generating hydrogen. The hydrogen generation rate greatly increases with pH, while the change of pH after the hydrogen generation reaction is negligibly low compared with that estimated assuming that OH^−^ ions are consumed for hydrogen generation. These results show that (i) the reacting species is OH^−^ ions and (ii) the concentration of OH^−^ ions remains unchanged during the hydrogen generation reaction. The unchanged pH indicates the reaction mechanism that OH^−^ ions are consumed in the initial reaction stage but electrons generated in the initial reaction stage are captured by water molecules, leading to generation of OH^−^ ions. Analysis of the relationship between the generated hydrogen volume and the reaction time confirms the reaction mechanism involving negative ions, i.e., OH^−^ ions. The surface reaction is the rate-determining step until the formation of monolayer Si–O bonds, and afterward, migration of OH^−^ ions through the oxide layer becomes rate-determining. The reaction of Si nanopowder to generate hydrogen stops when the thickness of a SiO_2_ layer on Si nanopowder reaches 4.8 nm. The reaction rate strongly depends on the crystallite size but not on the size of agglomerates of Si nanopowder.

## Electronic supplementary material

ESM 1 (DOCX 2.95 mb)
