# Supplementary material for: Hydrogen generation by reaction of Si nanopowder with neutral water
Source: J Nanopart Res. 2017 May 16;19(5):176. doi: 10.1007/s11051-017-3873-z (PMC5434163; doi:10.1007/s11051-017-3873-z)
Supplement: Supplementary file 1 — (DOCX 2.95 mb) [file 11051_2017_3873_MOESM1_ESM.docx]

Supplementary material

Hydrogen generation by reaction of Si nanopowder with neutral water

Yuki Kobayashi, Shinsuke Matsuda, Kentaro Imamura, and Hikaru Kobayashi*

The Institute of Scientific and Industrial Research, Osaka University, Osaka 567-0047, Japan

*E-mail: [h.kobayashi@sanken.osaka-u.ac.jp](mailto:h.kobayashi@sanken.osaka-u.ac.jp)

**Fig. S1**  XRD patterns of Si powders: (a) before milling; (b) after one-step milling; (c) after two-step milling. One-step milling was performed using 0.5 mm zirconia beads, and for two-step milling, 0.3 mm zirconia beads were used after use of 0.5 mm zirconia beads.

Fig. S1 shows the XRD patterns of Si powders before (pattern a) and after (patterns b and c) beads milling. X-ray diffraction (XRD) measurements were performed with a Rigaku RINT2500 diffractometer. All the observed peaks were attributable to Si crystals, and the intensity ratios of the observed diffraction peaks were nearly unchanged by beads milling. The Si(111) diffraction peak was the most dominant, and the (111) lattice constant was determined to be 0.31447 and 0.31717 nm, respectively, for one-step and two-step milling, which constants were slightly larger than that for bulk Si of 0.31346 nm. The widths of the XRD peaks were much larger after beads milling, indicating formation of nano-sized powder. From the width of the Si(111) diffraction peak, the average diameter, $D$*,* of Si nanopowder produced using the one-step (pattern b) and two-step (pattern c) milling methods was estimated to be 23.4 and 13.8 nm, respectively, using the Scherrer’s equation:

$$D=\frac{K\lambda}{Bcos\theta}$$

where $B$ is the full-width at half maximum of the diffraction peak, $\theta$ is the Bragg angle, $\lambda$ is the wavelength of X-ray, and $K$ is the Sherrer’s constant.

(b)

(a)

**Fig. S2** Volume distribution of the diameter of Si nanopowders fabricated using the following methods: (a) one-step milling method, (b) two-step milling method.

Fig. S2 shows the volume distribution of the diameter of Si nanopowers obtained from analysis of the shape of the Si(111) X-ray diffraction peak for Si nanopowders fabricated by the one-step (curve a) and two-step (curve b) milling methods. The mode diameter, i.e., diameter at the distribution maximum, for two-step milling of 5.8 nm is only slightly smaller than that for one-step milling of 6.6 nm. The median diameter (i.e., lines A and B by which the distribution profile is divided into the same two areas) for the one-step and two-step milling methods was 14.0 and 9.6 nm, respectively.

**Fig. S3** TEM (a) and SEM (b) micrographs of Si nanopowder fabricated by the one-step milling method.

Fig. S3 shows the transmission electron micrograph (TEM) and scanning electron micrograph (SEM) for Si nanopowder fabricated by the one-step bead milling method. The TEM micrograph was observed using a JEOL JEM-ARM200F microscope with the incident electron energy of 200 keV and observation of SEM micrograph was carried out using a JOEL JSM-6335F microscope. The TEM micrograph clearly shows that Si nanopower includes many Si nanocrystals with sizes less than 10 nm. On the other hand, the SEM micrograph demonstrates that Si nanopower consists of aggregates with sizes of ~100 nm.

**Fig. S4**  Volume distribution of the Si nanopowder aggregates produced with the following methods: (q) one-step milling method, (b) two-step milling method.

Fig. S4 shows the size distribution of aggregates of Si nanopowder fabricated using the one- and two-step beads milling methods obtained from the light scattering measurements using an Otsuka Electronics ELSZ-1000 apparatus. The diameters of Si aggregates produced using the one- and two-step milling methods are determined to be 201 and 192 nm, respectively, and the median diameters are 168 and 161 nm, respectively. The distribution profile of Si aggregates fabricated using the two-step milling method is shifted to smaller size direction only by 5~10 nm from that for the one-step milled Si nanopowder.
